# Engaging Patients in Atrial Fibrillation Management via Digital Health Technology: The Impact of Tailored Messaging

**DOI:** 10.19102/icrm.2020.110802

**Published:** 2020-08-15

**Authors:** Tammy Toscos, Amanda Coupe, Shauna Wagner, Ryan Ahmed, Amelia Roebuck, Mindy Flanagan, Michelle Drouin, Michael Mirro

**Affiliations:** ^1^Parkview Mirro Center for Research and Innovation, Health Services and Informatics Research Department, Parkview Health, Fort Wayne, IN 46845, USA

**Keywords:** Atrial fibrillation, consumer health informatics, digital health, medication adherence, tailored messaging

## Abstract

Patients with atrial fibrillation (AF) demonstrate persistent knowledge gaps regarding their condition and a substandard adherence to oral anticoagulant (OAC) medication, which contribute to thromboembolic stroke and other clot-related complications. Tailored patient education and medication reminders may help reduce these negative health outcomes. We sought to improve disease knowledge and medication adherence among a sample of AF patients using tailored education and nudges. The intervention leveraged three digital health technologies: a patient portal, an electronic-prescribing data feed, and a smart pill bottle. The content of the educational messaging, nudges, and cadence were tailored according to findings from our user-centered design studies and delivered via a patient portal (MyChart®; Epic Systems, Verona, WI, USA), with which participants were familiar. In a six-month randomized controlled trial with parallel groups, we used MyChart® to send educational messages and medication reminders according to a decision tree that emerged from our prior user-centered design studies. The intervention group demonstrated higher AF knowledge at study completion than the control group and more MyChart® logins throughout the trial, suggesting intervention uptake. Women were more adherent than men and patients diagnosed more than one year ago were more adherent than those with more recent diagnoses. The intervention and control group adherence rates were 93.1% and 89.5%, respectively; intervention effect was moderated by age, medication type, and prior MyChart® use. Within the intervention group, younger patients, those taking once-daily rivaroxaban, and those who were less active MyChart® users prior to the study benefited relative to their control group counterparts. Tailored educational and reminder messages contributed to increased adherence and disease knowledge among AF patients, though certain patient characteristics moderated the intervention’s effectiveness. Technology-based health interventions can be useful for older adults with effective tailoring and training.

## Introduction

AF is a common chronic cardiac arrhythmia that is increasing with the growing aging population and which is associated with a least a four- to fivefold rise in stroke risk.^1^ Although oral anticoagulants (OACs) decrease AF patients’ stroke risk, more than half of these individuals do not take them as prescribed,^2^ due in part to a lack of knowledge about their condition and treatment.^3,4^ In recent years, the development of direct OACs (DOACs), which require less monitoring and have a shorter half-life than warfarin, has prompted additional adherence concerns and rendered adherence more crucial.^5,6^ As such, researchers have called for improved patient education to lessen this treatment gap.^7,8^

Although multiple individual and systemic factors contribute to medication adherence,^9^ education also plays a key role.^10,11^ AF patients desire more education than they are currently given,^4,12^ while persistent knowledge gaps and low adherence illustrate that education should be a continual process in line with AF’s chronic nature.^3,4,13,14^ Current education may also not be appropriately tailored to patient needs or health literacy, even though this is important for understanding, application, and making behavior changes.^15–18^ As such, advances in technology, such as electronic health record (EHR) patient portals, provide an enhanced means of continual patient–provider communication and present an opportunity to tailor the content of such communication, for example by using information available within the EHR.^19^

Some prior research has examined educational interventions for AF patients. One-time educational sessions have been associated with higher warfarin adherence at six months^20^ and lower AF-related complications at one year.^21^ Other studies have assessed the impact of continual education interventions, including a combined health education and reminder calendar model that resulted in increased dabigatran adherence across one year^22^ and a smartphone app with clinical decision support and education that was found to increase adherence, AF knowledge, quality of life, and OAC satisfaction at one and three months.^23^ These studies, combined, illustrate the potential for increasing adherence and other positive outcomes via AF patient education. However, we identified an opportunity to present a continual education intervention via technology that this older patient population may already use in a health care context (ie, EHR patient portal) as well as to tailor this intervention based on patient preferences and characteristics.

### Present study objectives

In response to the opportunities identified in extant literature, we designed a multiphase study. In the first phase, as reported in our prior work,^4^ we used focus groups and online surveys to elicit preferences from AF patients regarding content, timing, and delivery of AF-related educational information and reminders. Based on these preferences, we then created an algorithm to deliver tailored educational messages via an EHR patient portal (MyChart®; Epic Systems, Verona, WI, USA). In the present study, we implemented this intervention, hypothesizing that it would increase OAC adherence and AF knowledge.

## Methods

### Participants and setting

Participants included adult outpatient cardiology patients at a Midwestern United States hospital who had been diagnosed with nonvalvular AF and prescribed an OAC. Participants were also required to have access to a computer and the Internet as well as to have a MyChart® account or report the willingness to create one.

**Figure 1:**
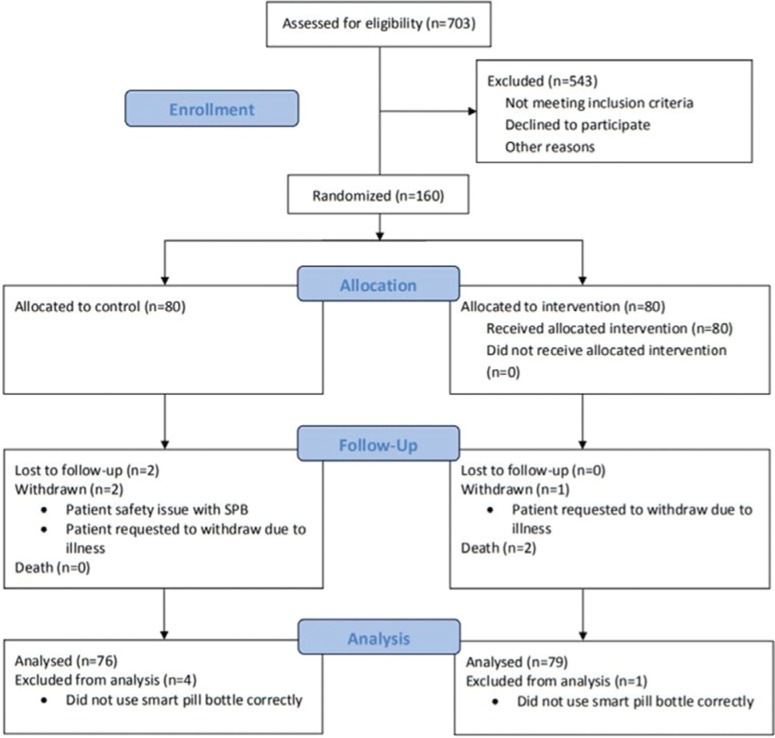
Study Consolidated Standards of Reporting Trials diagram. Process of participant screening, enrollment, group assignment, attrition, and inclusion in analyses.

Clinic staff and research team members identified 789 potential participants via a chart review, of which 703 were contacted by email, MyChart® message, letter, and/or telephone to screen for inclusion/exclusion criteria not available in the EHR. Recruitment was stratified to include equal numbers of individuals using DOACs (ie, rivaroxaban, apixaban, and edoxaban) and warfarin in both the intervention and control groups. Patients taking dabigatran could not be enrolled; we used an electronic pill bottle to track dosing and these pills must be stored in their original container. The enrollment target was 80 participants per group based on initial power calculations (power: 0.80; significance: 0.05). Ultimately, allowing for 20% attrition, a total of 160 patients were enrolled. Group assignment was randomized but not blinded. See **Figure 1** for a flowchart of the participant selection process.

Initially, participants met privately with a research team member for approximately one hour to provide written informed consent and complete surveys and training. Participants were compensated US$50 at both enrollment and completion on a reloadable debit card. Recruitment began in June 2017; the study concluded in November 2018. All study procedures were approved by the Parkview Health System Institutional Review Board and all procedures were performed in accordance with the standards established by the 1964 Declaration of Helsinki and its later amendments.

## Data collection and measures

### Survey data

Survey data were collected at baseline and exit. Paper-based surveys were administered to all participants during enrollment meetings. Paper surveys were mailed to the control group upon trial period completion; intervention group participants received links to secure, Health Insurance Portability and Accountability Act (HIPAA)-compliant web-based surveys hosted on SurveyMonkey (SVMK Inc., San Mateo, CA, USA) in their final study-related message.

***Participant characteristics.*** Various demographic information was collected, including age, gender, race, income, employment, education level, and time since diagnosis **(Table 1)**.

***Patient engagement.*** Patient engagement was assessed with the Patient Activation Measure (PAM),^24,25^ a validated self-report survey that operationalizes patient activation into four developmental stages: (1) believes importance of the patient role, (2) has confidence and the health-related knowledge required to act, (3) takes health-related actions, and (4) maintains actions despite stress. It included 10 items with a four-point Likert-type response set (1 = disagree strongly to 4 = agree strongly) that were scored and categorized into levels 1 through 4.

***Health literacy and knowledge of atrial fibrillation.*** Health literacy was assessed using the Newest Vital Sign (NVS) assessment,^26^ a nutrition label with six interpretive questions. The number of correct responses was totaled; four or more correct answers was considered to suggest the respondent had adequate health literacy. The AF Knowledge Scale^27^ included 11 items testing for the understanding of AF and its treatment. Correct responses were totaled. Potential scores ranged from zero to 11 points; we evenly divided them into a simple low/medium/high bracket (0–3, 4–7, or 8–11 points) to categorize the level of knowledge as such.

### Intervention uptake

MyChart® logins for the six months prior to and after study initiation were captured and dichotomized as low (0–12 logins) and high (13+ logins) based on the median (12 logins).

### Medication adherence

Medication adherence was tracked using AdhereTech’s Wireless Smart Pill Bottle (AdhereTech, New York, NY, USA), a HIPAA-compliant, Food and Drug Administration (FDA)-registered class I medical device that sends notifications when a user opens or fails to open the lid, based on dose schedule. In real-world use, these data would go to the user; however, for study purposes, they were sent to the study team, who then sent reminder messages to the intervention group (detailed below). Aside from using the smart pill bottle to store and dispense medication, the control group continued with standard care.

Participants were instructed to inform study staff of prescription/dosing changes as well as any temporary discontinuation of either their OAC or the bottle for any reason (eg, hospitalization, travel). For these situations, prescribed doses were adjusted accordingly for analyses.

### Intervention

**Figure 2:**
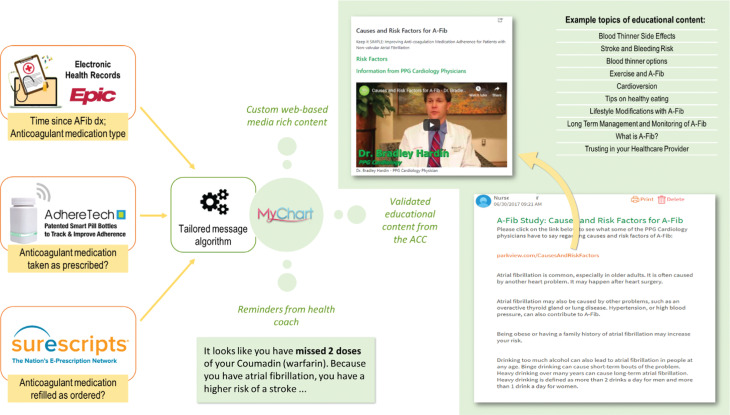
Intervention with sample message. Participants received educational and reminder messages based on information gathered from multiple digital health technologies. Example reminder text is shown, along with example educational topics and images of a sample educational message and its corresponding video.

**Figure 3:**
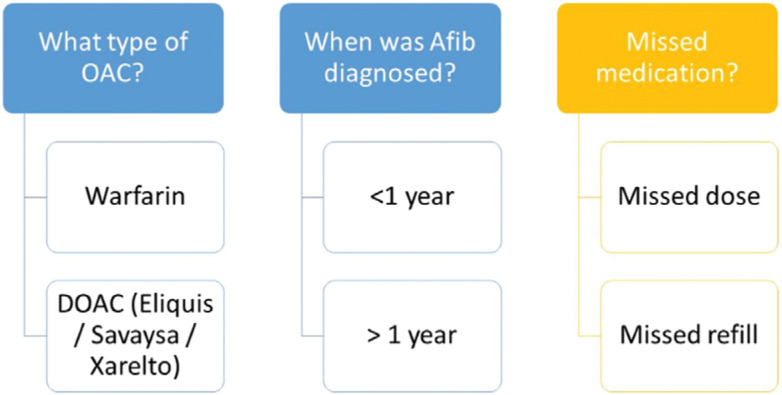
Messaging algorithm. Educational messages (blue) were tailored based on anticoagulant and time since diagnosis. Event-specific reminders (yellow) were sent when participants missed either anticoagulant doses or prescription refills.

Participants in the intervention group were sent educational messages and reminders via MyChart® throughout the six-month trial. See **Figures 2 and 3** for more information.

### Educational messages

Based on patient content preferences identified previously,^4^ the research team created 24 educational messages, using content from the American College of Cardiology’s patient education site, Cardiosmart (www.cardiosmart.org). Additionally, the study team created 14 videos featuring short interviews with four cardiologists and one pharmacist discussing topics surrounding AF and anticoagulation, together with patient testimonials; links to these videos were included in 14 corresponding messages.

### Reminders

Participants were sent a medication-specific reminder message upon missing one DOAC dose or two warfarin doses, as recorded by the bottle, with a follow-up message also sent upon missing a second DOAC dose or two additional warfarin doses. These messages included a reminder of the importance of OACs in stroke prevention, a suggestion to call the doctor about side effects or refills, and a prompt to email the study coordinator about bottle issues or routine changes that could cause inaccurate recordings. Participants received similar messages if they opened their bottle two or four extra times (depending on the medication being used) in one week.

SureScripts electronic prescribing information was used to track pharmacy refills. Participants were sent reminder messages upon being one week overdue for a refill, with a follow-up notice sent when two weeks overdue. These messages included the same suggestion to call one’s doctor with concerns and to notify the study team of routine changes or data inaccuracies (eg, receiving medication samples from their provider in lieu of renewing their prescription).

### Data analysis

Descriptive statistics were calculated for all measures, including means and standard deviations (SDs) for continuous variables and frequencies for categorical variables. The intervention was hypothesized to impact medication adherence; this was tested using generalized linear models. For these models, medication adherence was represented as the number of doses taken as a proportion of the prescribed number of doses. The rate of adherence was estimated in a generalized linear model with Poisson distribution using the log of total prescribed doses as an offset term in the linear predictor. A generalized linear model was tested that included (1) age, gender, employment status, income, education level, and time since diagnosis; (2) baseline PAM, AF knowledge, and NVS results; and (3) MyChart® use predicting medication adherence. These predictors were initially tested as main effects in separate models. Subsequently, for each model, the study group and interactions between each predictor and study group variable were entered, testing whether predictors moderated the effect of intervention on adherence. Participant OAC medication was included in all models as it was a stratification variable for sample selection (1 = warfarin, 2 = rivaroxaban, 3 = apixaban). The set of predictors and interaction terms that were significant were included in one combined model and significant effects were retained. For comparison, a negative binomial distribution was also used to estimate models. However, the model fit statistics [deviance and Pearson’s chi-squared (*χ*^2^) test] were not meaningfully improved; therefore, results generated using a Poisson distribution are presented.

As a check on intervention uptake, the following measures corresponding to the expected intervention impacts were tested for differences between study groups: (1) number of MyChart® patient portal logins and (2) AF knowledge. The number of MyChart® logins during the study period was tested for relation to the study group, prior MyChart® logins, and participant characteristics using a generalized linear model. Also, a generalized linear model assessed whether AF knowledge at study completion was related to the study group, baseline AF knowledge, or participant characteristics. Data analysis was completed using the SAS version 9.4 software program (SAS Institute, Cary, NC, USA).

## Results

In total, 160 participants were enrolled (n = 80 intervention and n = 80 control participants; 62.5% male; 99.4% white; mean ± SD age: 71.1 ± 8.5 years) **(Table 1)**. No significant demographic differences emerged between the groups. See **Figure 1** for details related to participant attrition and inclusion in the analyses.

### Intervention uptake and knowledge of atrial fibrillation

Prior to the study, each group had similar MyChart® login activity (mean ± SD: 18.6 ± 20.8 times for the control group; mean ± SD: 17.8 ± 18.8 times for the intervention group). However, during the study period, the rates differed significantly (*χ*^2^ = 62.04; p < 0.0001), with the intervention group (mean ± SD: 42.7 ± 37.0 times) conducting significantly more logins than the control group (mean ± SD: 15.9 ± 16.0 times). Additionally, the intervention group demonstrated greater AF knowledge at study completion than the control group when controlling for AF knowledge at baseline (*χ*^2^ = 6.66; p = 0.0099).

### Intervention impacts on medication adherence

As shown in **Table 2**, the final model included gender, age, time since diagnosis, medication type, prestudy MyChart® use, and study group. Males presented lower rates of medication adherence relative to females, while those diagnosed with AF more than one year prior to the study showed greater adherence to medication use than those who received their diagnoses more recently.

Overall, the intervention and control groups demonstrated medication adherence rates of 93.1% and 89.5%, respectively. In the main effects–only model, the intervention had a marginally significant effect on medication adherence (p = 0.08) **(Table 3)**. The moderating effects of age, medication type, and prestudy MyChart® use are illustrated in **Table 4**, which offers predicted values for medication adherence devised from the final fitted model. The moderated effect of intervention by age reveals that younger participants benefited more from the intervention in terms of adherence. Specifically, intervention participants at the mean age (71 years; z = −3.02; p = 0.003) and one SD below (63 years; z = −4.71; p < 0.0001) showed higher adherence relative to their control counterparts, whereas, at one SD above the mean age (79 years), there were no differences between the study groups. Type of medication moderated the effect of the intervention such that participants taking rivaroxaban had significantly higher adherence (z = −3.20; p = 0.001) in the intervention group than the control group; no differences in adherence emerged for other OAC types. Finally, participants with low MyChart® use prior to the study benefited more from the intervention, exhibiting higher adherence in comparison with among their control group counterparts (z = −3.61; p = 0.0003); this difference was not found among those with high MyChart® use prior to the study.

## Discussion

Our findings demonstrate positive outcomes of delivering a tailored messaging intervention to patients with AF. Though similar to prior work,^20–23^ our study focused on continued education via a health technology approach (EHR patient portal) with which patients were familiar. Relative to the control group, the intervention group participants logged into MyChart® significantly more often during the study period and had higher AF knowledge scale scores at exit. Although we cannot isolate the impact of the educational messages versus the medication reminders on adherence, the comparatively higher level of AF knowledge in the intervention group at exit suggests participants were reading and retaining the information they were given.

We also identified other variables related to adherence, including patient characteristics that moderated the effect of the intervention. The older adult participants in our sample (mean age: 71.1 years) were overall successfully engaged by this technology-based intervention. However, intervention group participants at the mean age and one SD below (ie, 71 and 63 years) showed higher adherence than their control group counterparts, while this effect disappeared by one SD above the mean (79 years). This underscores the importance of considering age differences and, potentially, individuals’ varied technological familiarity within the older adult age group and modifying the training accordingly.

In contrast with prior research suggesting that adherence decreases across time since diagnosis with a chronic condition,^28^ patients diagnosed more than one year prior to the study demonstrated greater adherence than those diagnosed more recently. This could be due to lived experiences, wherein patients diagnosed longer may have had more time to establish a routine, have had more contact with the health system, or have experienced an AF-related complication that motivated adherence; prior research suggests that higher-risk AF patients maintain higher adherence rates^9,29^ for these reasons.^6^ Still, this suggests an opportunity to tailor interventions to better engage newly-diagnosed AF patients, who often initially feel overwhelmed with information—another reason patients with recent diagnoses may have exhibited lower adherence.^4^

Additionally, as seen in some prior research,^6^ women demonstrated higher adherence rates than men. Lastly, we noted that patients taking rivaroxaban demonstrated greater adherence than those taking warfarin or apixaban, possibly because of the former’s once-daily dosing regimen.^30^ This illustrates the importance of considering medication type and dosing regimen when designing interventions to increase OAC adherence and evaluating their effectiveness.

### Limitations and future directions

Considered together, our outcomes and participant demographics suggest the presence of a potential volunteer bias and/or ceiling effect. Both the intervention and control groups demonstrated much higher OAC adherence rates than what is often reported among AF patient samples (93.1% and 89.5%, respectively, versus as low as 50%)^2^ and this discrepancy has been discussed in prior work.^31^ Perhaps, in a related fashion, the main effect of this intervention on medication adherence was not statistically significant. However, the intervention did show effects for participants with specific characteristics, providing a framework for future research.

The study sample was overwhelmingly white (99.4%) and educated (only 3.25% did not have a high school diploma/GED), limiting generalizability. AF patients with higher levels of information discomfort are less adherent^16^ and those with less education may have more negative attitudes toward health care^32^; these patients may be less likely to have volunteered for the study. Future research could attempt to limit volunteer bias and include a more ethnically and educationally diverse patient population by implementing a similar intervention into standard care.

Our sample also likely had higher health engagement and technological familiarity than the average AF patient, as patients without computer or Internet access and those who did not have or were unwilling to create a MyChart® account were not included in this study.^33^ Relatedly, intervention participants with high MyChart® usage prior to the study did not benefit relative to their control group counterparts, while less active users did, suggesting a potential ceiling effect.

As with nearly all methods of tracking medication adherence, there is some degree of accuracy limitation. We combined the smart pill bottle data with self-reports regarding events like medication holds or schedule disruptions to correct as many bottle-related inaccuracies as possible. Additionally, due to our use of the aforementioned bottle, we were unable to include patients taking dabigatran, as this medication must be stored in its original container. Further evaluation of the smart pill bottle’s research utility is detailed in our other work^34^ and the development of adherence technologies remains an important area of exploration.

We included a link to an online postsurvey in the final study-related MyChart® message sent to intervention group participants. Meanwhile, we mailed paper-based surveys to the control group, who had not been contacted via MyChart® during the study; we felt this would be a more reliable means of contact. These different administration methods are linked with group membership; thus, any related effect cannot be separated.

Lastly, although this was not within the intended scope of the present study, we recognize that clinical outcomes (eg, lower hospitalization rates, less thromboembolic events) are an important consideration when gauging the efficacy of interventions for medication adherence among AF patients; future research should explore this when building upon these initial findings.

## Conclusions

We present a framework for a tailored messaging intervention for AF patients taking OACs. We demonstrated the potential of combining three digital health technologies (patient portal, an electronic prescribing data feed, and a smart pill bottle) to deliver tailored educational messages and nudges that were designed in partnership with patients living with AF. Those who received educational messages and time-sensitive reminders through the study intervention demonstrated greater AF knowledge and higher rates of accessing the patient portal than those who did not. Intervention group participants with certain characteristics (eg, younger age, lower prestudy patient portal use, once-daily dose OAC medication) showed better OAC adherence than their control group counterparts and there was a nonstatistically significant trend toward increased adherence in the intervention group overall. Given the prevalence and severity of AF, coupled with persistent AF knowledge gaps despite efforts being made toward improving patient education, our findings underscore the importance and interrelated nature of health education and medication adherence. While our findings demonstrate the efficacy of a high-fidelity prototype technology solution for AF education and OAC reminder intervention within a relatively homogenous study population, future research should focus on refining tailored messaging and context-sensitive delivery through a user-centered design approach that includes individuals of a more diverse racial, educational, and socioeconomic background.

## Figures and Tables

**Table 1:** Participant Characteristics

Characteristic	Number Valid	Category	%
Gender	160	Female	37.50
Male	62.50
Age (years)	160	41–50	1.25
		51–60	11.25
		61–70	31.25
		71–80	46.25
		81–90	9.38
		91–100	0.63
Race	154	White or Caucasian	99.4
		Asian	0.65
		Black or African-American	0
		Hispanic or Latino	0
		American Indian or Alaska Native	0
		Native Hawaiian or other Pacific Islander	0
Employment status	158	Employed full-time	13.92
		Employed part-time	5.06
		Unemployed	1.90
		Disabled/unable to work	5.70
		Retired	73.42
Education level	154	Did not graduate high school	3.25
		High-school graduate/GED	21.43
		Trade/some college	29.87
		College graduate	25.32
		Postgraduate degree	20.13
Annual income	149	< $20,000	5.37
		$20,000–40,000	22.82
		$40,001–60,000	24.16
		$60,001–80,000	24.83
		$80,001–100,000	9.40
		> $100,000	13.42
Household occupants	158	Lives alone	16.46
		Lives with a spouse/partner	77.85
		Lives with a family member	5.06
		Lives with a friend	0.63
PAM	155	Level 1	10.32
		Level 2	29.68
		Level 3	49.68
		Level 4	10.32
Health literacy (NVS)	158	High likelihood of limited literacy	6.33
		Possibility of limited literacy	16.46
		Adequate literacy	77.22

NVS: Newest Vital Sign; PAM: Patient Activation Measure.

**Table 2:** Summary Results from Generalized Linear Modeling Predicting Medication Adherence (n = 155)

Parameter	Estimate	Standard Error	Wald 95% Confidence Limits		p > χ^2^
Intercept	−0.12	0.07	−0.25	0.008	0.07
Male (versus female)	−0.03	0.01	−0.05	−0.01	0.0047
Age	0.0001	0.0009	−0.002	0.002	0.90
AF diagnosis at > 1 year (versus < 1 year)	0.06	0.02	0.03	0.10	< 0.0001
Warfarin (versus apixaban)	0.02	0.02	−0.02	0.05	0.41
Rivaroxaban (versus apixaban)	0.04	0.02	−0.002	0.09	0.06
Low MyChart use (versus high)	0.01	0.02	−0.02	0.04	0.45
Control (versus intervention)	−0.32	0.09	−0.50	−0.13	0.0006
Study group × age	0.005	0.001	0.002	0.007	0.0002
Study group × education (warfarin)	−0.02	0.02	−0.07	0.03	0.37
Study group × medication (rivaroxaban)	−0.10	0.03	−0.17	−0.03	0.0033
Study group × MyChart use	−0.05	0.02	−0.09	−0.002	0.04
**Goodness of Fit**	**Value**	**DF**	**Value/DF**		
Deviance	207.52	143	1.45		
Pearson’s chi-squared	198.37	143	1.39		

AF: atrial fibrillation; DF: degrees of freedom.

**Table 3:** Summary Results from Main Effects–only Generalized Linear Modeling Predicting Medication Adherence (n = 155)

Parameter	Estimate	Standard Error	Wald 95% Confidence Limits		p > χ^2^
Intercept	−0.25	0.05	−0.35	−0.16	< 0.001
Male (versus female)	−0.03	0.01	−0.05	−0.005	0.02
Age	0.002	0.0006	0.001	0.004	< 0.001
AF diagnosis at > 1 year (versus < 1 year)	0.06	0.02	0.02	0.09	< 0.001
Warfarin (versus apixaban)	0.01	0.01	−0.01	0.03	0.44
Rivaroxaban (versus apixaban)	0.004	0.02	−0.03	0.04	0.83
Low MyChart use (versus high)	−0.01	0.01	−0.03	0.01	0.25
Control (versus intervention)	−0.02	0.01	−0.04	0.002	0.08
**Goodness of Fit**	**Value**	**DF**	**Value/DF**		
Deviance	235.35	147	1.60		
Pearson’s chi-squared	219.74	147	1.49		

AF: atrial fibrillation; DF: degrees of freedom.

**Table 4:** Predicted Values from Generalized Linear Modeling Predicting Medication Adherence (n = 155)

Parameter	Predicted Value	Standard Error of Mean	χ^2^	p > χ^2^
Gender				
Male	89.8	0.009	7.97	0.0048
Female	92.8	0.01		
Age				
63 years	89.4	0.009	14.92	0.0001
71 years	91.3	0.008		
79 years	93.1	0.009		
AF diagnosis				
< 1 year	88.4	0.007	15.38	< 0.0001
; 1 year	94.3	0.014		
Medication				
Warfarin	91.8	0.01	0.45	0.80
Rivaroxaban	90.7	0.01		
Apixaban	91.4	0.009		
MyChart use				
Low	90.8	0.009	0.97	0.32
High	91.8	0.009		
Study group				
Control	89.5	0.01	16.58	< 0.0001
Intervention	93.1	0.009		
Study group × age:			13.36	0.0002
Control × 63 years	86.0	0.01		
Control × 71 years	89.5	0.01		
Control × 79 years	93.0	0.01		
Intervention × 63 years	93.0	0.01		
Intervention × 71 years	93.1	0.009		
Intervention × 79 years	93.2	0.01		
Study group × medication			8.68	0.01
Control × warfarin	90.9	0.01		
Control × rivaroxaban	86.3	0.02		
Control × apixaban	91.5	0.01		
Intervention × warfarin	92.7	0.01		
Intervention × rivaroxaban	95.3	0.02		
Intervention × apixaban	91.3	0.01		
Study Group × MyChart use			4.21	0.04
Control × low use	88.0	0.01		
Control × high use	91.0	0.01		
Intervention × low use	93.6	0.01		
Intervention × high use	92.5	0.01		

AF = atrial fibrillation.
